# NLRP1 variant M1184V decreases inflammasome activation in the context of DPP9 inhibition and asthma severity

**DOI:** 10.1016/j.jaci.2020.12.636

**Published:** 2021-06

**Authors:** Jonas Moecking, Pawat Laohamonthonkul, Katelyn Chalker, Marquitta J. White, Cassandra R. Harapas, Chien-Hsiung Yu, Sophia Davidson, Katja Hrovat-Schaale, Donglei Hu, Celeste Eng, Scott Huntsman, Dale J. Calleja, Jay C. Horvat, Phil M. Hansbro, Robert J.J. O’Donoghue, Jenny P. Ting, Esteban G. Burchard, Matthias Geyer, Motti Gerlic, Seth L. Masters

**Affiliations:** a Inflammation Division, The Walter and Eliza Hall Institute of Medical Research, Parkville, Australia; b Department of Medical Biology, University of Melbourne, Parkville, Australia; c the Institute of Structural Biology, University of Bonn, Venusberg-Campus 1, Bonn, Germany; d Department of Medicine, University of California, San Francisco, Calif; e the Priority Research Centre for Healthy Lungs, Hunter Medical Research Institute, New Lambton, Australia; f University of Newcastle, Callaghan, Australia; g Centre for Inflammation, Centenary Institute, Sydney, Australia; h Faculty of Science, University of Technology Sydney, Ultimo, Australia; i Department of Pharmacology and Therapeutics, University of Melbourne, Melbourne, Australia; j Department of Microbiology and Immunology, University of North Carolina, Chapel Hill, NC; k Department of Bioengineering & Therapeutic Sciences, University of California, San Francisco, San Francisco, Calif; l Department of Clinical Microbiology and Immunology, Sackler Faculty of Medicine, Tel Aviv University, Tel Aviv, Israel

**Keywords:** NLRP1, inflammasome, asthma, DPP9, SNP, ASC, Apoptosis-associated speck-like protein containing a CARD, BAL, Bronchoalveolar lavage, BMI, Body mass index, DPP9, Dipeptidyl peptidase 9, GALA II, Genes-Environment & Admixture in Latino Americans II, NLRP, Nucleotide-binding oligomerization domain-like receptor containing a pyrin domain, OVA, Ovalbumin, SAGE II, Study of African Americans, Asthma, Genes, and Environments II, SNP, Single nucleotide polymorphism, WT, Wild-type

## Abstract

**Background:**

NLRP1 is an innate immune sensor that can form cytoplasmic inflammasome complexes. Polymorphisms in *NLRP1* are linked to asthma; however, there is currently no functional or mechanistic explanation for this.

**Objective:**

We sought to clarify the role of *NLRP1* in asthma pathogenesis.

**Methods:**

Results from the GALA II cohort study were used to identify a link between *NLRP1* and asthma in Mexican Americans. *In vitro* and *in vivo* models for NLRP1 activation were applied to investigate the role of this inflammasome in asthma at the molecular level.

**Results:**

We document the association of an *NLRP1* haplotype with asthma for which the single nucleotide polymorphism rs11651270 (M1184V) individually is the most significant. Surprisingly, M1184V increases NLRP1 activation in the context of N-terminal destabilization, but decreases NLRP1 activation on dipeptidyl peptidase 9 inhibition. *In vitro* studies demonstrate that M1184V increases binding to dipeptidyl peptidase 9, which can account for its inhibitory role in this context. In addition, *in vivo* data from a mouse model of airway inflammation reveal a protective role for NLRP1 inflammasome activation reducing eosinophilia in this setting.

**Conclusions:**

Linking our *in vitro* and *in vivo* results, we found that the NLRP1 variant M1184V reduces inflammasome activation in the context of dipeptidyl peptidase 9 inhibition and could thereby increase asthma severity. Our studies may have implications for the treatment of asthma in patients carrying this variant of NLRP1.

## Graphical abstract

**Figure:**
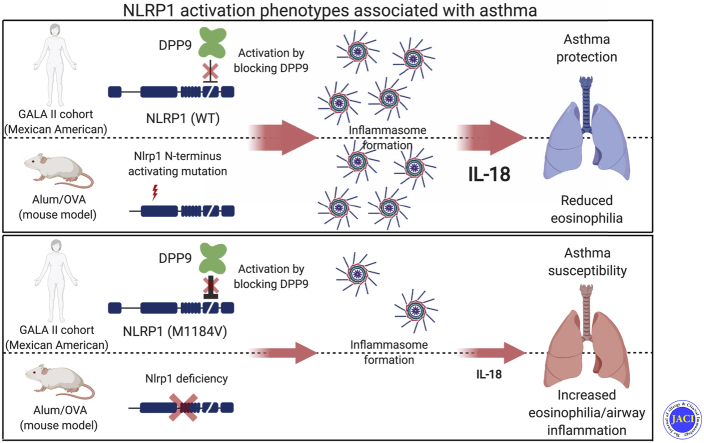

Asthma is a common chronic respiratory disease that according to World Health Organization estimates affects 235 million people worldwide. It typically occurs in genetically predisposed individuals after inappropriate immune activation caused by antigen exposure, such as viral infections and allergens, and subsequent epithelial damage.^1^^,^^2^ Patients suffer from airway inflammation, obstruction, and remodeling, as well as airway hyperresponsiveness.^3^ Because asthma is an incurable disease, patients require continued treatment and management.^4^

Release of proinflammatory cytokines from the IL-1 family (IL-1β, IL-18, IL-33) is a hallmark of asthmatic inflammation. IL-1β and IL-33 activate T_H_2/T_H_17 cells and ILC2/3 cells, leading to the release of proinflammatory cytokines (IL-4, IL-5, IL-9, IL-13, IL-17A).^5^, ^6^, ^7^ Subsequently, eosinophils, neutrophils, mast cells, and T_H_2 cells infiltrate the lung to trigger inflammation and tissue damage.^8^^,^^9^ The other IL-1 family member, IL-18, is also linked to asthma, but with conflicting effects. On the one hand, it is described to inhibit T_H_2-cell development together with IL-12, resulting in reduced airway hyperresponsiveness in a mouse model of asthma.^10^ Moreover, significantly increased serum levels of IL-18 were found in patients during acute asthma episodes.^11^ On the other hand, reduced levels of IL-18 were found in the bronchoalveolar lavage (BAL) fluid of patients with asthma compared with healthy controls.^12^ To date, the exact role of IL-18 in asthma pathogenesis is not entirely clear.^13^

Inflammasomes form an important part of the innate immune system and are potent activators of procaspase-1. As such, they are able to induce the release of mature IL-1β and IL-18 as well as a rapid form of cell death called pyroptosis.^14^ The current model suggests that upon expression NOD-like receptors are localized in the cytosol of the cell as monomers. Immediately after activation, they assemble into disc-like oligomers, providing a platform for the adaptor protein ASC (apoptosis-associated speck-like protein containing a CARD). In turn, ASC assembles into large filaments and recruits procaspase-1, ultimately leading to activation of procaspase-1 by self-cleavage. Active caspase-1 can process the pro forms of IL-1β and IL-18 into their active forms and thus induce inflammation.^14^^,^^15^ Furthermore, active caspase-1 cleaves gasdermin D, which in turn forms membrane pores inducing pyroptosis.^16^

NLRP1 was the first inflammasome described and has been shown to be expressed in various tissues including the respiratory epithelium, as opposed to NLRP3, which is primarily expressed in the hematopoietic compartment.^17^^,^^18^ The 166-kDa NLRP1 protein also differs from other nucleotide-binding oligomerization domain-like receptors containing a pyrin domain (NLRPs) in its domain composition. The NLRP1 N-terminus is formed by a common tripartite domain architecture that is shared by all NLRP proteins, composed of a PYD, a NACHT domain, and leucine-rich repeats. However, an additional FIIND and CARD domain on the C-terminus is unique to NLRP1.^19^ Autolytic proteolysis within the FIIND domain is a strict requirement for NLRP1 activity.^20^^,^^21^ A common single nucleotide polymorphism (SNP; rs11651270) resulting in the amino acid substitution methionine 1184 to valine (M1184V) was described to increase cleavage in the FIIND domain and has been associated with genetic predisposition to asthma.^21^^,^^22^ Furthermore, SNPs in *NLRP1* were found to be involved in other diseases, such as vitiligo-associated autoimmune diseases.^23^ Several mutations in the *NLRP1* gene locus were identified to lead to constitutive inflammasome activation, causing skin autoinflammatory syndromes.^24^^,^^25^ These patient-derived mutations helped to understand the mechanistic role of NLRP1 PYD and leucine-rich repeat in keeping the protein in an autoinhibited conformation.^24^ The fact that the NLRP1 PYD is autoinhibitory differentiates this inflammasome from other members of the NLRP family, which require their PYD to directly bind the adaptor protein ASC and thus enable downstream activation of procaspase-1.^26^ In contrast, it is the C-terminal CARD domain of NLRP1 that triggers inflammasome activation.^21^^,^^24^

To date, the exact mechanism by which NLRP1 is activated through pathogens is not fully understood. However, *Toxoplasma gondii* has been demonstrated to activate NLRP1 in mice and rats, and genetic variants in human NLRP1 increase susceptibility to congenital toxoplasmosis.^27^, ^28^, ^29^, ^30^ Dense granule proteins of the parasite have been identified to be essential for triggering NLRP1-dependent pyroptosis in rats.^31^ Lethal toxin from *Bacillus anthracis* cleaves and thereby activates certain alleles of mouse and rat Nlrp1.^32^^,^^33^ Recently, different pathogen enzymes were identified to induce N-terminal degradation of mouse NLRP1b. Cleavage within the FIIND domain provided, this degradation process led to activation of the NLRP1 inflammasome.^34^ In addition, it has been shown that inhibition of the negative regulator dipeptidyl peptidase 9 (DPP9), which binds to the FIIND domain, leads to activation of NLRP1 in humans and mice.^35^^,^^36^ Inhibition of DPP9 leads to a dissociation of the peptidase from NLRP1 and results in N-terminal degradation and finally activation of the inflammasome.^37^^,^^38^ Moreover, 1 of the human mutations causing skin inflammatory symptoms and associated arthritis was identified to be located within the FIIND domain (P1214R).^25^ This mutation was shown to activate NLRP1 by preventing DPP9 association with NLRP1.^36^

Here, we confirm that the M1184V mutation within the FIIND domain of NLRP1 is associated with increased asthma severity. Surprisingly, we find that activation of NLRP1 M1184V *in vitro* via N-terminal–destabilizing mutations has divergent effects compared with activation via DPP9 inhibition. In addition, genetic manipulation of *Nlrp1* in mouse models of asthma shows that *Nlrp1* deficiency exacerbates asthma models. Combined with the *in vitro* data, this aligns with a causative effect of M1184V in asthma to decrease NLRP1 activation in the context of DPP9 inhibition.

## Methods

### Plasmids and mutagenesis

Viral constructs including pRP-hASC-RFP (retroviral construct) and pTRIPz-NLRP1-HA-T2A-GFP-IRES-Puro (second-generation lentiviral construct) were used to establish cell lines with stable expression of the transgenes. For immunoprecipitation, NLRP1 constructs were expressed from a pCIG2 vector backbone with a C-terminal 3xFLAG-tag and eGFP was expressed from an IRES on the same vector. Mutagenesis was carried out using the QuikChange Lightning Site-Directed Mutagenesis Kit (Agilent, Santa Clara, Calif) following the manufacturer’s instructions. Successful mutation was confirmed by sequencing.

### Cell culture

HEK293T cells were cultured at 37°C and 5% CO_2_ in Dulbecco modified Eagle medium (Thermo Fisher Scientific, Waltham, Mass) supplemented with 10% FBS, 0.1% (wt/vol) streptomycin, and 100 U/mL penicillin. FBS was heat-inactivated for 30 minutes at 55°C before supplementation. Continuous cultures were monitored for absence of mycoplasma by PCR.

### Generation of stable cell lines

Production of viral particles for stable expression of transgenes was achieved by transfecting HEK293T with packaging plasmids pGag-pol and VSV-G along with the transgene hASC-RFP for retrovial system and psPAX2 and pMD2.G along with the transgene NLRP1-HA-T2A-GFP-IRES-Puro for second-generation lentiviral system, respectively. Plasmids were transfected into HEK293T cells, seeded at 3 × 10^6^ cells in 10-cm^2^ dishes 1 day before the transfection was performed. Medium of the transfected cells was replaced 16 hours posttransfection. The viral supernatant was collected and filtered through a 0.45-μm filter at 24 hours and 48 hours after medium change. The filtered viral supernatant was then added directly to HEK293T and incubated for 24 hours before the viral supernatant was entirely removed.

Successful retroviral transduction of hASC-RFP cells was confirmed and cells were isolated by fluorescence-activated cell sorting for RFP^+^ nonspecking cells. These cells were used as a parental cell line for the subsequent lentiviral transduction of NLRP1-HA-T2A-GFP-IRES-Puro. Following the lentiviral transduction of NLRP1-HA-T2A-GFP-IRES-Puro, the cells were subjected to 5 μg/mL puromycin selection before sorting for GFP^−^ nonspecking cells. Expression of the NLRP1-T2A-GFP transgene was induced using 1 μg/mL doxycycline (Sigma Aldrich, St Louis, Mo), and the expression was confirmed by flow cytometry and western blot analysis.

### ASC speck assay

A total of 3.5 × 10^4^ HEK293T cells stably expressing human ASC-RFP and stably carrying the doxycycline-inducible wild-type (WT) or mutant human NLRP1 transgene were seeded in a flat-bottom 96-well tissue culture plate. The cells were seeded in the presence of 1 μg/mL of doxycycline to induce the expression of the transgene. Cells were harvested and analyzed for speck formation 12 hours postinduction by flow cytometry.

For Talabostat treatment, the medium was replaced at 16 hours after doxycycline induction with medium containing increasing concentration of Talabostat (MedChemExpress HY-13233A) from 0.125 μM to 1 μM. The highest equivalent amount of dimethyl sulfoxide was used as a control. Following Talabostat treatment, cells were incubated for 6 hours at 37°C. Cells were then harvested and analyzed for speck formation by flow cytometry on a BD Bioscience LSR Fortessa X-20 as described previously.^39^

### Immunoprecipitation

A total of 2.5 × 10^5^ HEK293T cells were transfected with 500 ng of WT or mutant NLRP1 (IRES-GFP). Eighteen hours posttransfection, cells were washed once with 1×DPBS and harvested in NP40 lysis buffer (1% NP40 (vol/vol), 10% glycerol (vol/vol), 20 nM Tris-HCl, 150 mM NaCl, 1 mM ethyleneglycol-bis-(β-aminoethylether)-N,N,N',N'-tetraacetic acid, 10 mM NaPPi, 5 mM NaF, 1 mM Na_3_VO_4_, 1 mM phenylmethylsulfonyl fluoride) freshly supplemented with 1× cOmplete protease inhibitor cocktail (Roche, Basel, Switzerland). After lysing cells for 20 minutes on ice, cell debris was spun down and the supernatant was collected. Immunoprecipitation was performed using anti–FLAG-M2-agarose resin (Sigma) for 4 hours or overnight at 4°C. Beads were washed 3 times with lysis buffer before elution by boiling in SDS sample buffer for 10 minutes. Immunoblots were prepared using 4% to 12% gradient gels (Novex, Invitrogen, Carlsbad, Calif) and subsequently transferred to a PVDF membrane. Membranes were blocked in PBS/tween 20 with 5% skim milk for 60 minutes at room temperature and probed overnight at 4°C. The following antibodies were used: aNLRP1: AL176 (AdipoGen, San Diego, Calif), aDPP9: ab42080 (Abcam, Cambridge, UK), aFLAG: 9H1 (in-house), and aActin: sc47778 (SCBT).

### Mice

WT, Nlrp1^−/−^, Il1r^−/−^, Il1r^−/−^Neut1^m/m^, Il1r^−/−^Il18^−/−^, and Il1r^−/−^Il18^−/−^Neut1^m/m^ mice described previously^40^ were analyzed between age 1.5 and 3 months. Animal experiments complied with the regulations set by the Walter and Eliza Hall Institute of Medical Research Animal Ethics Committee.

### Asthma model

Mice were immunized with sensitization solution (50% (vol/vol) aluminum hydroxide [Sigma], 250 μg/mL ovalbumin [lyophilized chicken egg white albumin] [Sigma], 10% (vol/vol) type II water, and 40% (vol/vol) normal saline [WEHI Media Department, Australia]; 200 μL/mouse) via intraperitoneal injection on days 1 and 14. On days 27, 28, and 29, mice were challenged via nebulization (inhalation) of challenge solution (MTPBS [WEHI Media Department], 5% (wt/vol) ovalbumin [Sigma]) for 20 minutes. Mice were sacrificed and prepared for assessment of asthma pathology on day 30.

### Isolation and preparation of organs and fluids

The BAL fluid was collected by performing a tracheotomy on the mice before flushing the lungs with 3 × 500 μL flow buffer (MTPBS [WEHI Media Department], 1% FBS, 2.5 mM EDTA). Next, lungs were collected and the left lobe retained in 10% buffered formalin for histology. Lungs were diced with scissors and digested at room temperature for approximately 30 minutes in digestion buffer (RPMI [WEHI Media Department], 1 mg/mL collagenase III [Worthington, Lakewood, NJ], 0.4 U dispase, 1 μg/mL dnase) with mixing. Single-cell suspensions were obtained by passing the digested lungs through a 70-μm sieve. Cells were collected by centrifugation. BAL fluid supernatants were collected and stored at −20°C for later analysis, and red cells lysed in red cell removal buffer (WEHI Media Department) for 1 minute at room temperature. Cells were then washed and resuspended in flow buffer. Cells were incubated with CD16/32 Fc block for 20 minutes before incubation with cell surface staining combination. Cells were then incubated with fixation buffer (eBioscience), permeabilization buffer (eBioscience, San Diego, Calif), and intracellular antibodies, and washed between steps. Cells were resuspended in 200 μL of flow buffer. Spleen single stain controls were included for all experiments. All centrifugations were performed at 1300*g* for 5 minutes at 4°C, and all incubations were on ice for 20 minutes.

### Flow cytometry

**Fig E1:**
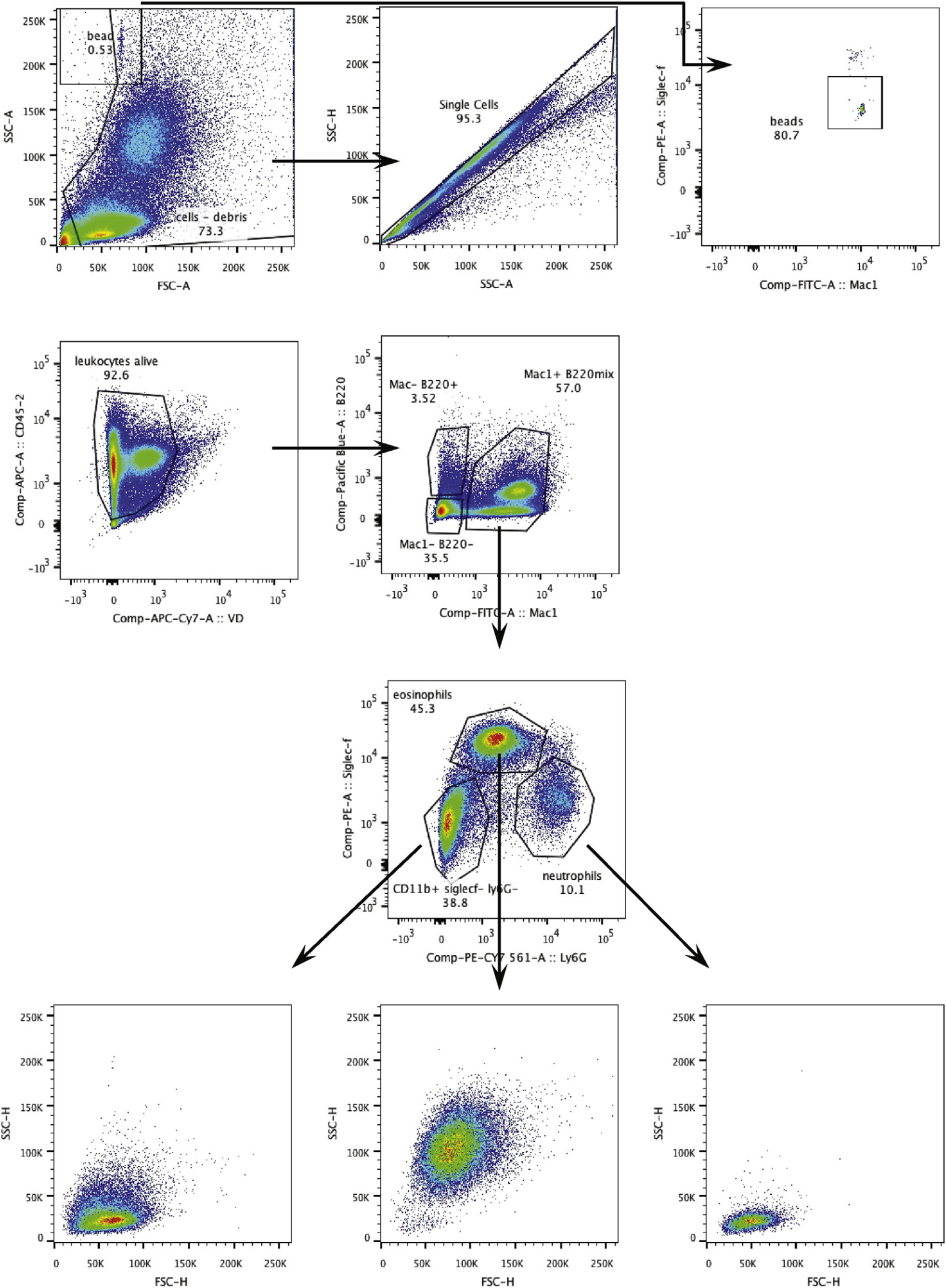
Gating strategy for inflammatory cell identification. Debris was gated out, and beads were identified for quantification. Beads were further defined to remove contaminating cells. Doublets were removed and live leukocytes identified before gating for granulocyte populations. First, granulocytes were defined as CD45.2^+^, B220^−^, or CD19^−^, CD11b^+^ cells and afterwards further separated into eosinophils or neutrophils on the basis of Siglec-F^+^ Ly6G^−^ or Siglec-F^−^ Ly6G^hi^, respectively. Macrophages/monocytes were defined as Siglec-F^-^, Ly6G^−^, and CD11b^+^. *FSC-A*, Forward scatter-area; *FSC-H*, forward scatter-height; *SSC-A*, side scatter-area; *SSC-H*, side scatter-height.

**Fig E2:**
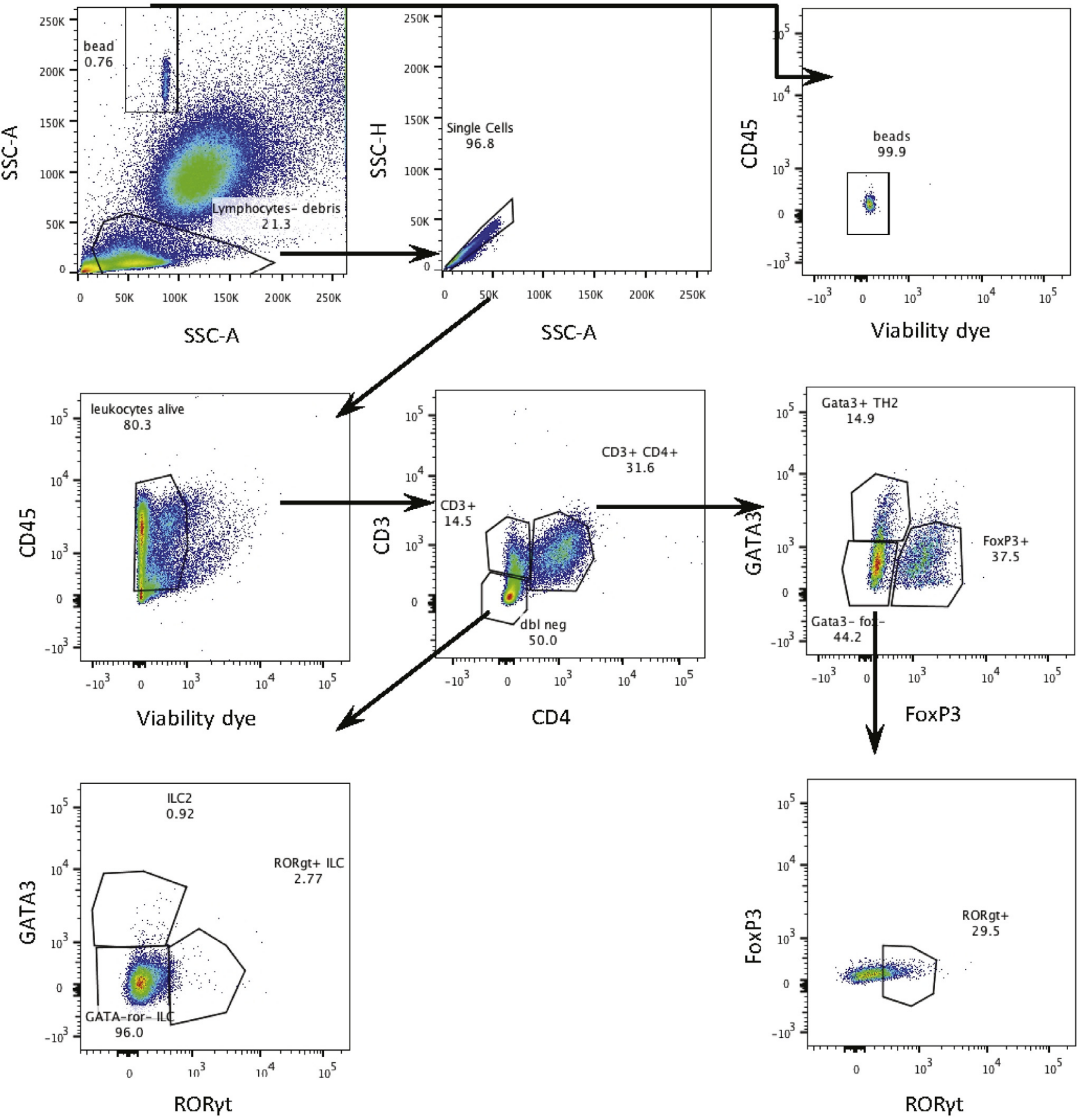
Gating strategy for T-cell subset and ILC identification. Debris was gated out, and beads were identified for quantification. Beads were further defined to remove contaminating cells. Doublets were removed, and live T lymphocytes were defined as CD45^+^CD3^+^CD4^+^. T lymphocytes were further classified as regulatory T cells, T_H_2 cells, or T_H_17 cells by expression of FoxP3, GATA3, or RORγt, respectively. ILCs were defined as CD45^+^CD3^−^CD4^−^ cells stratified into ILC2 or ILC3 by expression of GATA3 or RORγt, respectively. *ILC,* Innate lymphoid cell; *ILC2*, type 2 innate lymphoid cell; *ILC3*, type 3 innate lymphoid cell; *SSC-A*, side scatter-area; *SSC-H*, side scatter-height.

Flow cytometry was performed on a BD Bioscience LSR Fortessa cell analyzer, and analysis was performed using FlowJo software (BD, San Jose, Calif). Granulocytes were defined as CD45.2^+^, B220^−^, or CD19^−^, CD11b^+^ cells and further defined as eosinophils or neutrophils on the basis of Siglec-F^+^ Ly6G^−^ or Siglec-F^−^ Ly6G^hi^, respectively. Macrophages/monocytes were defined as Siglec-F^−^ and Ly6G^−^ CD11b^+^. T lymphocytes were defined as CD45^+^ CD3^+^ CD4^+^ B220 cells and further classified as regulatory T lymphocytes, T_H_2, or T_H_17 by expression of FoxP3, GATA3, or RORγt, respectively. Innate lymphoid cells were defined as CD45^+^ lineage negative (CD3^−^/CD4^−^) cells and further stratified into type 2 Innate lymphoid cells or type 3 Innate lymphoid cells by expression of GATA3 or RORγt, respectively. Single stain controls were included for all flow cytometry experiments to facilitate appropriate compensation. Representative dot plots for the gating strategies are shown in Figs E1 and E2 in this article’s Online Repository at www.jacionline.org.

### Histopathology

Lungs were collected in 10% buffered formalin. Sections were stained with hematoxylin and eosin, periodic acid-Schiff, and toluidine blue for detection of inflammatory cells, mucus production, and mast cells.

### Multiplex ELISA

Multiplex ELISAs were performed using the BioPlex Pro Mouse Cytokine Kit (BioRad, Hercules, Calif) following the manufacturer’s instructions. Absorbance was recorded at 450 nm using a microplate reader.

### Statistics

Data were analyzed using the Prism software (GraphPad, San Diego, Calif). Comparison of data was performed using a Student *t* test or an ANOVA followed by a paired *t* test. Values are displayed as mean ± SEM.

### Genes-environment and Admixture in Latino Americans II and Study of African Americans, Asthma, Genes, and Environments II cohort studies

The Genes-environments and Admixture in Latino Americans II (GALA II) study and the Study of African Americans, Asthma, Genes, and Environments II (SAGE II) study are 2 clinic-based multicenter asthma case-control studies, conducted using identical protocols and questionnaires, to examine the complex network of genetic and environmental factors contributing to asthma prevalence and severity among Latino and African American children.^41^^,^^42^ Full descriptions of the GALA II study and SAGE II study protocols and recruitment, including inclusion and exclusion criteria, have been previously given in detail.^41^^,^^42^ Briefly, asthma cases and controls were recruited from community centers and clinics in the mainland United States and Puerto Rico (2006-present). Individuals were eligible to participate if they were aged 8 to 21 years and identified all 4 grandparents as Latino (GALA II) or African American (SAGE II). Participants were excluded if they had (1) 10 or more pack-years of smoking; (2) any smoking within 1 year of recruitment date; (3) pregnancy in the third trimester; or (4) history of 1 of the following conditions: sickle cell disease, cystic fibrosis, sarcoidosis, cerebral palsy, or heart or chest surgery. Demographic data, including medical history and environmental exposure information, were collected from participants at study enrollment. All local institutional review boards of participating recruitment sites approved the study, and all participants (or parents of participants younger than 18 years) provided written informed consent.

Asthma case/control status was determined by physician assessment at study enrollment. Age- and sex-specific body mass index (BMI) percentiles were calculated as previously described and used to assign BMI categories.^43^ For subjects 20 years and older, BMI categories were defined as follows: nonobese (BMI < 30) and obese (BMI ≥ 30). For subjects younger than 20 years, BMI categories were defined as follows: nonobese (BMI percentile < 95) and obese (BMI percentile ≥ 95).

### Study population

Mexican American subjects from the GALA II study cohort with complete demographic data (age, sex, asthma status, obesity status) and available NLRP1 SNP were used as a discovery data set in the current study (n = 905). Puerto Rican participants from the GALA II study (n = 1418) and African American participants from the SAGE study (n= 1256) were later examined to determine whether associations between NLRP1 variants and asthma (both single-variant and haplotype effects) present in the discovery data set were also present in these non-Mexican populations. Study demographics for all individuals in the discovery data set (n = 905 Mexican Americans) and the 2 replication data sets (n = 1418 Puerto Ricans; n = 1256 African Americans) are presented in Table E1, Table E2, Table E3 in this article’s Online Repository at www.jacionline.org. All descriptive statistics presented in Table E1, Table E2, Table E3 were generated using the R statistical software program base package.

### NLRP1 genotyping

Blood samples were collected from GALA II and SAGE II participants at study enrollment for DNA analysis. Axiom Genome-Wide LAT 1 array (Affymetrix, Santa Clara, Calif, dbGaP phs000921.v1.p1) was used for genotyping. SNPs were excluded if they failed the manufacturer’s quality control, had genotyping call rates below 95%, and had a deviation from Hardy-Weinberg equilibrium (*P* < 10^−6^) within controls. Additional SNP genotypes were imputed using the Michigan Imputation Server. The National Heart, Lung, and Blood Institute Trans-Omics for Precision Medicine program data (freeze 5) were used as the imputation reference panel.^44^ Imputed SNPs were excluded from the data set if *R*^2^ was below 0.3. Genotypes of several NLRP1 SNPs, identified by previous studies as functionally relevant in relation to immunologic diseases,^45^ were then extracted from the total genotype data (see Table E4 in this article’s Online Repository at www.jacionline.org) for downstream single-variant and haplotype analyses. Principal-component analysis using genotyped data for all participants was performed using the PLINK 2.0 software platform.^46^ The first 3 principal components were included in downstream association analyses to account for hidden substructure in the data set. Genotype data for GALA II study participants are available on dbGaP under accession number phs001274, and genotype data for SAGE II study participants are available on Dryad (https://datadryad.org/stash/share/20Ma3IthxRaK5sxbTNHGpOMGeDnJVFTPxDIxECTh2is).

### Single-variant analyses

We used logistic regression to assess the relationship between each variant and asthma susceptibility, separately. All regression models were adjusted for age, sex, obesity status, and the first 3 principal components (generated as described in the previous subsection). We also calculated allele frequencies for each variant in participants with and without asthma, separately and combined (see Table E5, Table E6, Table E7 in this article’s Online Repository at www.jacionline.org). All single-variant genetic analyses were performed using the PLINK 2.0 software platform.^46^

### Multivariant (haplotype) analyses

NLRP1 variant haplotype analyses were performed using the Haplo.stats R software package.^47^ Haplo.stats is a statistical program optimized to assess the relationship between a specified haplotype and case/control status in unrelated individuals, when haplotype phase is unknown, using regression-based analyses. We identified 5 NLRP1 haplotypes, containing a total of 14 SNPs, previously defined by Levandowski et al,^45^ and shown by the authors to be significantly associated with vitiligo and other autoimmune disorders. Haplotype definition and haplotype frequencies in our study population are presented in Table E10, Table E11, Table E8, Table E9 in this article’s Online Repository at www.jacionline.org. The most commonly occurring haplotype, referred to as haplotype 1, in both asthma cases and controls was selected as the reference haplotype for regression-based analyses performed in Haplo.stats. Presented odds ratios for haplotype effects describe the effect of the specified haplotype on asthma status as compared with haplotype 1 (Table E10, Table E11, Table E8, Table E9).

**Table I:** *NLRP1* haplotypes associated with asthma status in Mexican American children from the GALA II study

	R1366C	rs6502867	V1241L	M1184T	M1184V	M1119V	V1059M	T995I	T878M	T782S	T246S	L155H	rs2670660	rs8182352	Odds ratio	95% CI	*P* value
Haplotype 1	G	T	C	A	T	T	C	G	G	G	G	A	A	T	Reference haplotype		
Haplotype 1C	G	C	C	A	T	T	C	G	G	G	G	A	A	T	1.46	1.05-2.02	.02
**Haplotype 2A**	**G**	**T**	**C**	**A**	**C**	**T**	**T**	**G**	**G**	**G**	**G**	**T**	**G**	**C**	**1.69**	**1.18-2.43**	**.004**
Haplotype 2B	G	T	C	G	C	T	T	G	G	G	G	T	G	C	1.35	1.01-1.79	.04
Haplotype 3	A	C	G	A	C	C	C	A	A	C	C	T	G	C	1.02	0.42-2.45	.97

Haplotypes significantly associated with asthma status, after correction for multiple testing, are highlighted in boldface.

### Multiple testing correction for single-variant and multivariant (haplotype) analyses

We used the Bonferroni method to control the familywise error rate and correct for multiple testing.^48^ Significance thresholds derived using the Bonferroni equation can also produce false negatives. To minimize type II error in our study, in addition to significant association thresholds, we generated a more liberal suggestive association threshold.^43^^,^^49^ For multivariant analyses, the effective number of tests was defined as the number of haplotypes assessed. Bonferroni familywise error rate thresholds of 0.05 and 0.1 were used to generate significant and suggestive association thresholds, respectively.^50^ The suggestive and significant *P*-value thresholds for single-variant analyses (3 variants) were .033 and .017, respectively. The suggestive and significant *P*-value thresholds for the multivariant analyses (4 haplotypes) were .025 and .0125, respectively.

## Results

### NLRP1 variant M1184V is associated with genetic predisposition to asthma

In a discovery cohort of 905 Mexican American children with and without asthma from the GALA II study, we assessed the effect of select *NLRP1* SNPs on asthma susceptibility. Descriptive characteristics for all discovery study subjects are summarized in Table E1. Demographic variables were assessed for significant differences between asthma cases and controls. Significant differences were found for age (*P* = 2.20 x 10^−16^), sex (*P* = .007), and obesity status (*P* = .01).

A previous study by Levandowski et al^45^ identified significant *NLRP1* haplotype effects associated with vitiligo and other immunologic conditions. We assessed the impact of these same haplotypes on asthma status in our study population. Regression-based haplotype analysis revealed 1 *NLRP1* haplotype significantly associated with asthma status, compared with the most common/reference haplotype (Table E10, Table E11, Table E8, Table E9). This haplotype, haplotype 2A, remained significantly associated with asthma after correction for multiple testing (odds ratio, 1.69; *P* = .004). Genotypes for all *NLRP1* SNPs included in haplotype 2A are listed in Table E8.

We then performed single-variant association testing, using logistic regression, to assess the impact of the 3 nonsynonymous *NLRP1* polymorphisms in haplotype 2A—L155H (rs12150220), V1059M (rs2301582), and M1184V (rs11651270)—on asthma status in our study population. Each regression analysis was adjusted for age, sex, obesity status, and the first 3 principal components. Two *NLRP1* variants, V1059M and M1184V, were associated with asthma status in our study; however, only 1 variant, M1184V, remained suggestively associated with asthma after correction for multiple testing (*P* = .02; Table E10, Table E11, Table E8, Table E9). Specifically, increased copies of the C allele of M1184V were associated with increased asthma susceptibility (odds ratio, 1.28; *P* = .02).

**Table II:** NLRP1 variants associated with asthma status in Mexican American children from the GALA II study

Variant	rs ID	Effect allele	Effect allele frequency	Odds ratio	95% CI	*P* value
L155H	rs12150220	T	0.42	1.20	0.97-1.49	.09
V1059M	rs2301582	T	0.40	1.28	1.03-1.59	.03
**M1184V**	**rs11651270**	**C**	**0.45**	**1.28**	**1.03-1.59**	**.02**

Variants significantly associated with asthma status after correction for multiple testing are highlighted in boldface.

After completing haplotype and single-variant analysis in our Mexican American data set, we attempted to further validate our findings in 2 independent data sets of Puerto Rican and African American children (see Tables E2 and E3). Neither the significant 2B haplotype effect nor the suggestive single locus effect at M1184V was present in either of our validation data sets (see Table E12, Table E13, Table E14, Table E15 in this article’s Online Repository at www.jacionline.org). Further assessment revealed differing allele and haplotype frequencies among the 3 populations, which may account, in part, for the lack of replication of significant and suggestive associations found in Mexican Americans.

### Activation of NLRP1 is reduced by M1184V in the context of DPP9 inhibition

**Fig 1:**
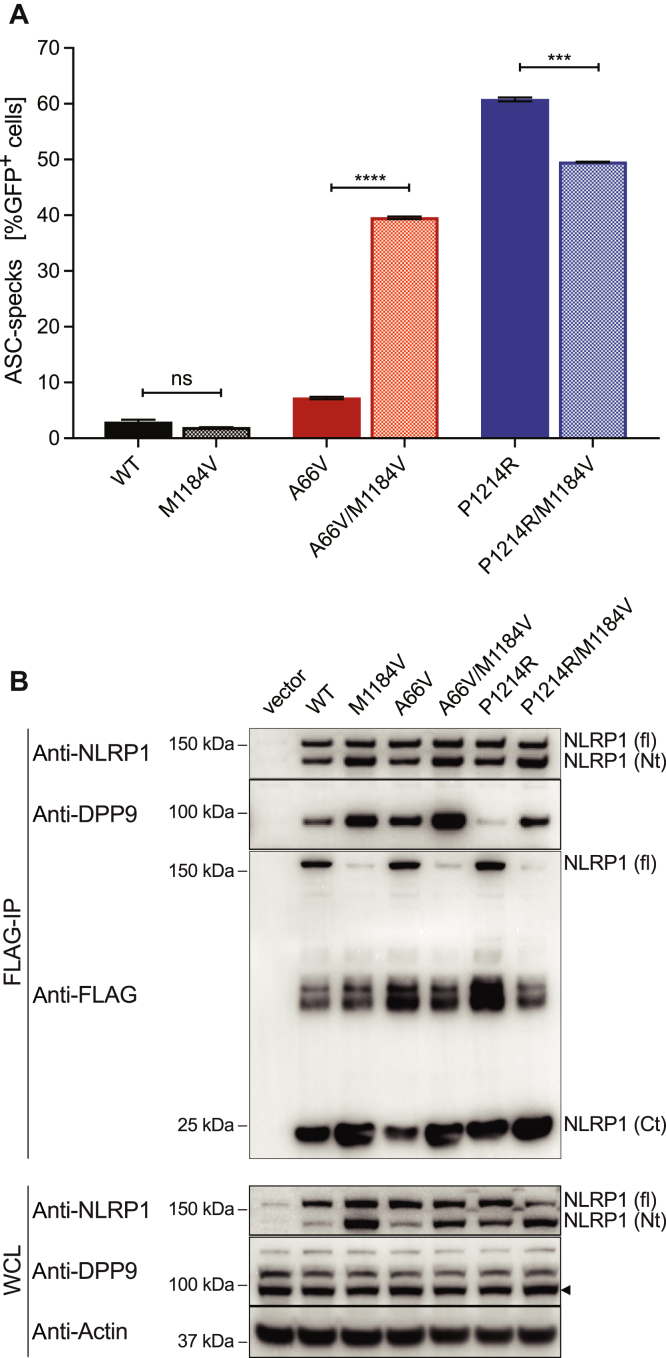
M1184V has divergent effects on NLRP1 activation due to altered DPP9 binding. **A,** A total of 3.5 × 10^4^ HEK 293T cells stably expressing a human ASC-RFP fusion protein were stably reconstituted with dox-inducible NLRP1-T2A-GFP (WT or indicated mutant). Cells were harvested 12 hours after doxycycline induction and analyzed for ASC speck formation by flow cytometric measurement. **B,** A total of 2.5 × 10^5^ HEK293T cells were transfected with 500 ng of a vector control or a plasmid encoding human NLRP1-3xFLAG (WT or indicated mutant). Eighteen hours posttransfection, cells were harvested, and immunoprecipitation was performed for analysis of protein expression by western blot. Data in Fig 1, *A*, are 3 biological replicates, representative of 3 independent experiments. *Ct*, C-terminal fragment; *fl*, NLRP1 full-length; *GFP*, green fluorescent protein; *ns*, nonsignificant; *Nt*, N-terminal fragment; *WCL*, whole cell lysate. Data are mean ± SEM. *P* values were calculated using unpaired *t* test between 2 groups. ∗∗∗*P* < .001. ∗∗∗∗*P* < .0001.

**Fig E3:**
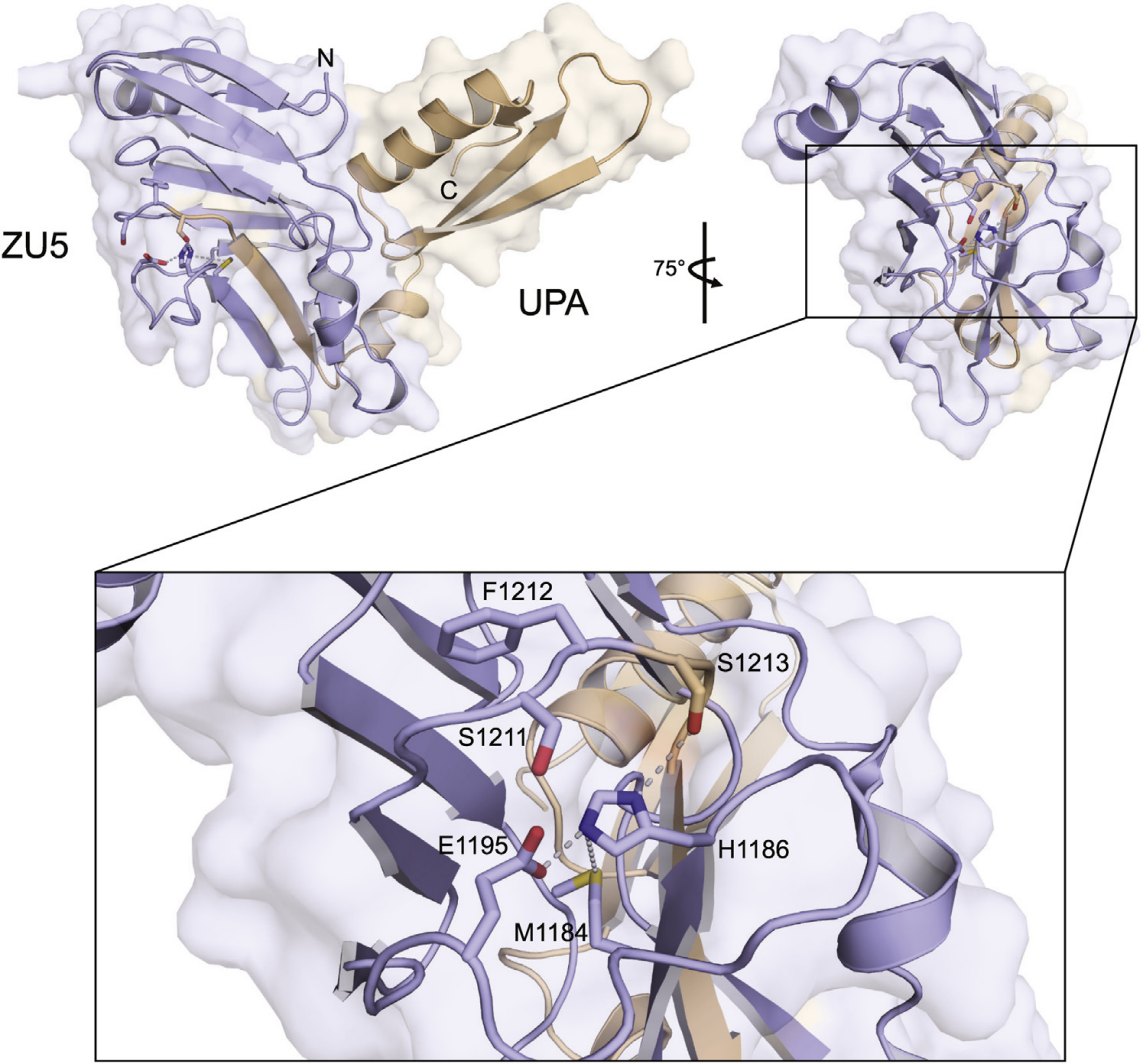
Homology model of the NLRP1 FIIND domain. The model of the NLRP1 FIIND domain (residues 1084-1278) was generated using Swiss model.^E1^ Secondary structure elements are shown in cartoon representation. Amino acid side chains of the cleavage site residues S1211 and F1212 as well as the side chains of the proposed catalytic triad S1213, H1186, and E1195 and the methionine affected by the polymorphism (M1184) are shown in stick representation. M1184 is not in direct proximity of the cleavage site but near the catalytic H1186. Potentially, substitution of M1184 affects cleavage by changing the positioning of the catalytic histidine, allowing it to induce cleavage more efficiently. Images and distance measurements were generated using PyMOL. The structure of the cytoplasmic domain of Unc5b (PDB: 3G5B)^E2^ served as a template for structure modeling.

The NLRP1 M1184V variant has been reported to increase FIIND domain cleavage in a previous study.^21^ Analysis of a homology model showing the ZU5 and part of the UPA subdomains of NLRP1 FIIND suggests that the respective methionine residue is located in proximity to the proposed catalytic triad consisting of amino acid residues S1213, H1186, and E1195 (see Fig E3 in this article’s Online Repository at www.jacionline.org). These residues were previously described to be directly involved in facilitating autolytic proteolysis between F1212 and S1213.^20^^,^^21^ Exchanging methionine 1184 to valine could alter the positioning of the catalytic residues relative to each other, thereby allowing increased proteolysis. However, the exact molecular mechanism of how substitution of methionine 1184 affects proteolytic processing remains unclear. In addition, the effect of M1184V has not yet been examined in the context of NLRP1 stimulation, either by activating mutations or by DPP9 inhibition. To investigate the functional effect of M1184V on inflammasome activation, we reconstituted NLRP1 in HEK293T cells stably expressing ASC-RFP. ASC speck formation was used as a measure for NLRP1 inflammasome activation and was quantified by flow cytometric analysis as previously described.^39^ NLRP1 WT and M1184V showed similar baseline levels of ASC speck formation (Fig 1, *A*). Western blot analysis for NLRP1 full-length and N-terminal cleavage fragments (anti-NLRP1), as well as the NLRP1 C-terminal cleavage fragment (anti-Flag), confirmed increased FIIND domain cleavage by M1184V (Fig 1, *B*).

NLRP1 inflammasome activation was triggered by introducing recently reported patient mutations into the WT and M1184V NLRP1 constructs. The A66V mutation was described to activate NLRP1 by destabilizing its PYD.^24^ As expected, introduction of this mutation into NLRP1 WT resulted in increased ASC speck formation. In the context of increased FIIND domain cleavage, induced by the M1184V polymorphism, this effect was enhanced as a further accumulation of ASC specks was detected (Fig 1, *A*). Increased FIIND domain cleavage for NLRP1 A66V/M1184V was confirmed by western blot analysis (Fig 1, *B*). Introduction of a different monogenic autoinflammatory disease mutation (P1214R), described to activate NLRP1 through a loss of DPP9 binding,^25^^,^^36^ also resulted in a strong increase in ASC speck formation. Interestingly, combining this mutation with M1184V reduced NLRP1 activity significantly, as quantified by ASC speck formation (Fig 1, *A*). Western blot analysis of the P1214R variant did not show a large increase in the C-terminal fragment, and combining P1214R with M1184V did not result in a further increase in C-terminal cleavage.

Thus, we concluded that increased cleavage of NLRP1 for the M1184V variant liberates more of the active C-terminal fragment, when in combination with mutations that destabilize the N-terminus, accounting for increased inflammasome activation. However, this cannot explain the decreased inflammasome activation observed when M1184V is present in conjunction with the P1214R mutation. Given that P1214R activates NLRP1 by destabilizing the interaction with DPP9, we performed immunoprecipitations to see whether DPP9 binding was altered because of M1184V. Indeed, DPP9 binding was abolished by P1214R (Fig 1, *B*). Compellingly, the M1184V variant increased binding to DPP9, even in the presence of P1214R, explaining how it inhibits NLRP1 activity in this context. As reported previously, A66V had no effect on DPP9 binding,^36^ and combination with M1184V also increased binding to DPP9 (Fig 1, *B*). Therefore, when the N-terminus of NLRP1 is destabilized, inflammasome activation proceeds regardless of DPP9 binding.

**Fig 2:**
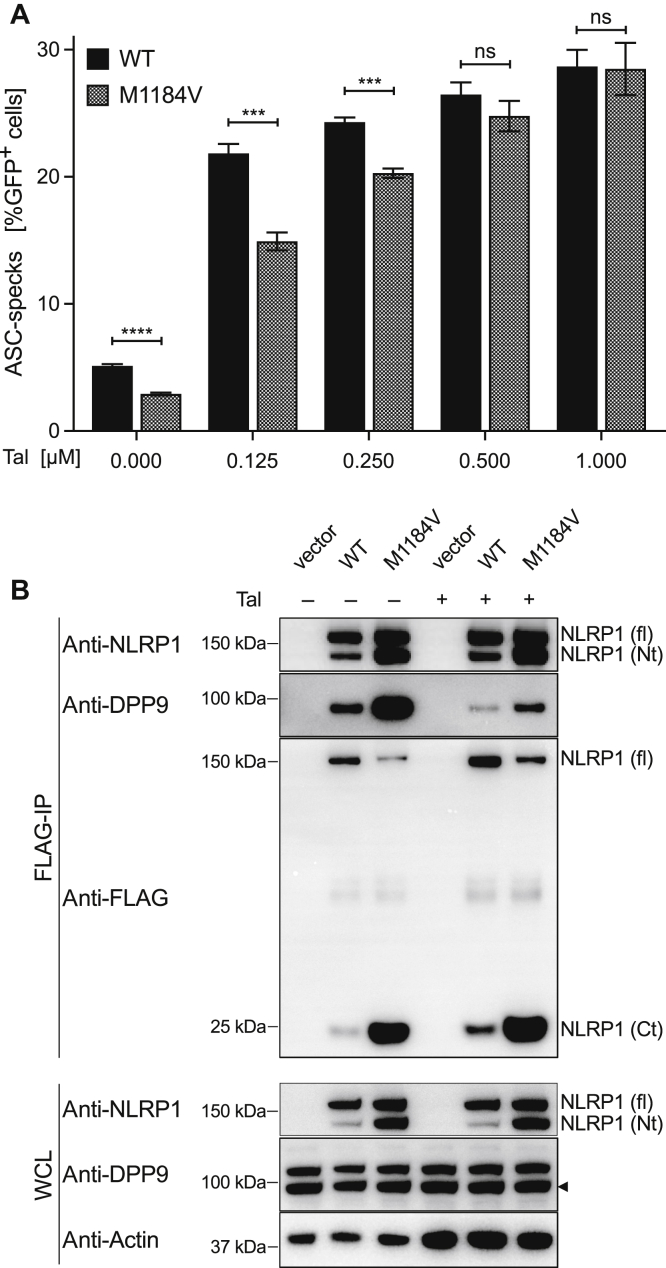
Activation of NLRP1 by Talabostat is reduced for M1184V because of enhanced DPP9 binding. **A,** A total of 3.5 × 10^4^ HEK293T cells stably expressing a human ASC-RFP fusion protein were stably reconstituted with dox-inducible NLRP1-T2A-GFP (WT or M1184V). Sixteen hours after doxycycline treatment, cells were stimulated with fresh medium containing increasing concentrations of Talabostat or the highest equivalent amount of dimethyl sulfoxide and incubated for 6 hours. Cells were harvested, and ASC speck formation analysis was performed by flow cytometry. **B,** A total of 2.5 × 10^5^ HEK293T cells were transfected with 500 ng of a vector control or NLRP1-3xFLAG (WT or M1184V). Eighteen hours posttransfection, cells were stimulated with 2 μM Talabostat or an equivalent amount of dimethyl sulfoxide as described in Fig 2, *A*. Following the treatment, cells were harvested, and immunoprecipitation was performed for protein expression by western blot. Data in Fig 2, *A*, are 3 biological replicates, representative of 3 independent experiments. Data in Fig 2, *A*, were pooled from 3 independent experiments. *Ct*, C-terminal fragment; *fl*, NLRP1 full-length; *GFP*, green fluorescent protein; *ns*, nonsignificant; *Nt*, N-terminal fragment; *WCL*, whole cell lystae. Data are mean ± SEM. *P* values were calculated using unpaired *t* test between 2 groups. ∗∗∗*P* < .001. ∗∗∗∗*P* < .0001.

To independently confirm the inhibitory effect of M1184V on NLRP1 activation induced by loss of DPP9 binding, we made use of the DPP9 inhibitor Talabostat. Consistent with the findings described for NLRP1 M1184V/P1214R, we found that activation by Talabostat was inhibited for NLRP1 M1184V, however only at lower thresholds of activation (Fig 2, *A*). Increased DPP9 binding in NLRP1 M1184V after Talabostat stimulation was also confirmed by immunoprecipitation (Fig 2, *B*). These results indicate that NLRP1 activation induced by impaired DPP9 binding is reversed by the M1184V variant, which stabilizes the interaction with DPP9.

### Nlrp1^−/−^ mice show increased eosinophil infiltration in asthma model

**Fig E4:**
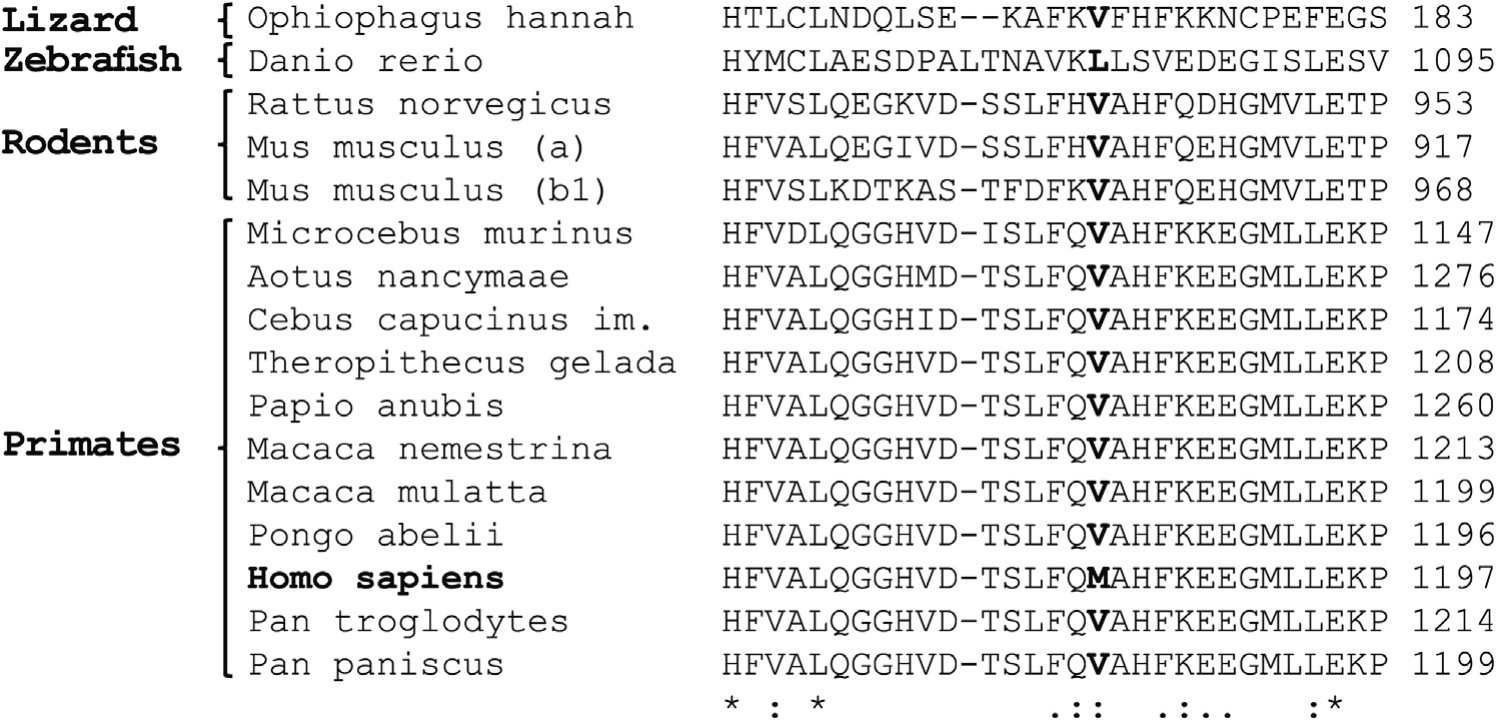
Sequence alignment of NLRP1 from different species. Sequence alignment of the amino acid sequence of NLRP1 from different organisms. Sequences were taken from NCBI. Multiple sequence alignment was performed using the Clustal Omega online tool with default settings.^E3^ Methionine 1184 in human NLRP1 and corresponding residues in other species are highlighted. Stars/dots in the bottom line indicate similarity (dots) or identity (stars) of the amino acids in the respective position.

To better understand the role of Nlrp1 in asthma, we used an alum/ovalbumin (OVA) mouse model. Noteworthy, mice variants of Nlrp1 were reported to naturally carry a valine at the position corresponding to the human M1184.^21^ We confirmed this finding by multiple sequence alignment of the amino acid sequence of NLRP1 in different organisms. Interestingly, we found that within the analyzed sequences, including sequences from different primate species, only humans have methionine as the canonical amino acid at position 1184 (see Fig E4 in this article’s Online Repository at www.jacionline.org).

**Fig 3:**
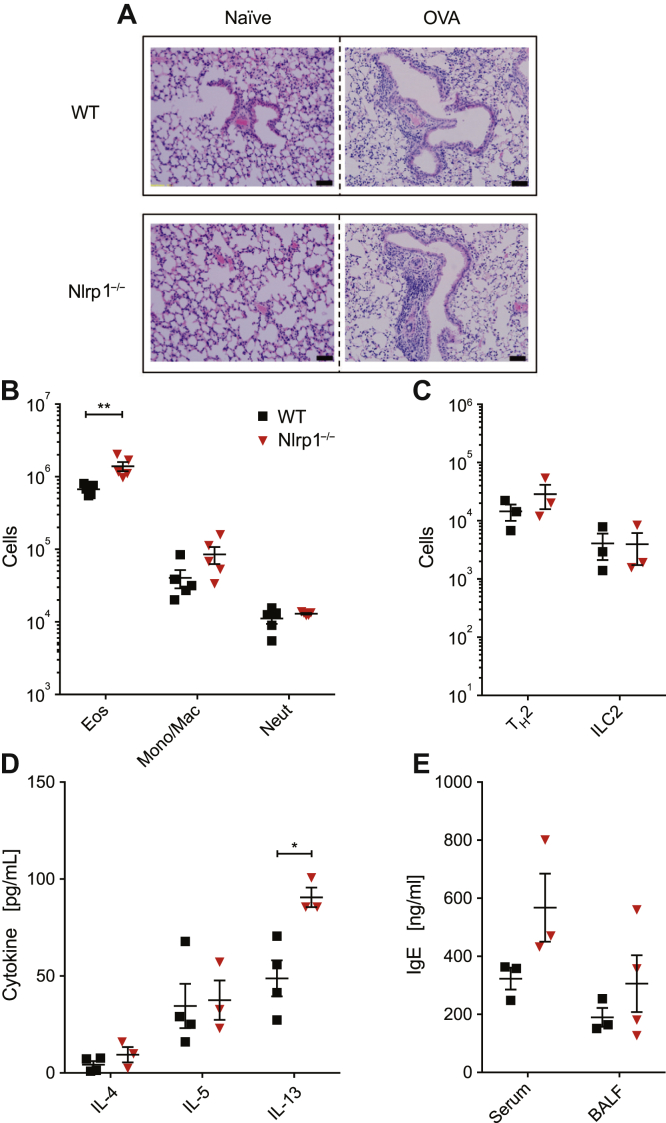
OVA-treated *Nlrp1*^*−/−*^ mice show increased lung eosinophilia and elevated IL-13 levels. **A,** Representative hematoxylin and eosin histology of left-lung lobe from WT and *Nlrp1*^*−/−*^ mice show no overt differences, either before or after OVA treatment. (**B**) Numbers of eosinophils (Eos), monocytes/macrophages (Mono/Mac), and neutrophils (Neut), and of (**C**) T_H_2 and ILC2 cells in the lungs of OVA-treated WT and *Nlrp1*^*−/−*^ mice. **D,** Cytokine levels in the BAL fluid of OVA-treated WT and *Nlrp1*^*−/−*^ mice. **E,** Serum and BAL fluid IgE levels of OVA-treated WT and *Nlrp1*^*−/−*^ mice. *BALF*, BAL fluid; *ILC2*, type 2 innate lymphoid cell. Data are mean ± SEM. Representative of 3 independent experiments. *P* values were calculated using unpaired *t* test between 2 groups. ∗*P* < .05. ∗∗*P* < .01.

Airway inflammation in mice was assessed by comparing immune cell infiltration into the lung and cytokine levels of WT and *Nlrp1*^*−/−*^ mice. Histological comparison of lung sections of WT and *Nlrp1*^*−/−*^ mice revealed no overt difference. *Nlrp1*^*−/−*^ mice showed no pathology at baseline (Fig 3, *A*), and both groups developed alveolitis and showed immune cell infiltration into the airways and lung vessels following alum/OVA treatment. Immune cell infiltration into the lung was measured by flow cytometric analysis of the lungs after BAL of alum/OVA-treated mice. Interestingly, *Nlrp1*^*−/−*^ mice showed increased eosinophilia compared with WT mice, suggesting a protective effect of NLRP1 in asthma (Fig 3, *B*). Levels of monocytes/macrophages, neutrophils, T_H_2 cells, and type 2 innate lymphoid cells were comparable for both WT and *Nlrp1*^*−/−*^ mice (Fig 3, *B* and *C*). A protective effect of Nlrp1 in the asthma model was also indicated by an increase in IL-13 in the BAL fluid of *Nlrp1*^*−/−*^ mice (Fig 3, *D*). IL-4 and IL-5 levels were comparable for WT and *Nlrp1*^*−/−*^ mice (Fig 3, *D*). Furthermore, IgE levels were comparable in the serum and BAL fluid of WT and *Nlrp1*^*−/−*^ mice (Fig 3, *E*).

### Active Nlrp1 protects against asthma model in mice independently of IL-1

**Fig 4:**
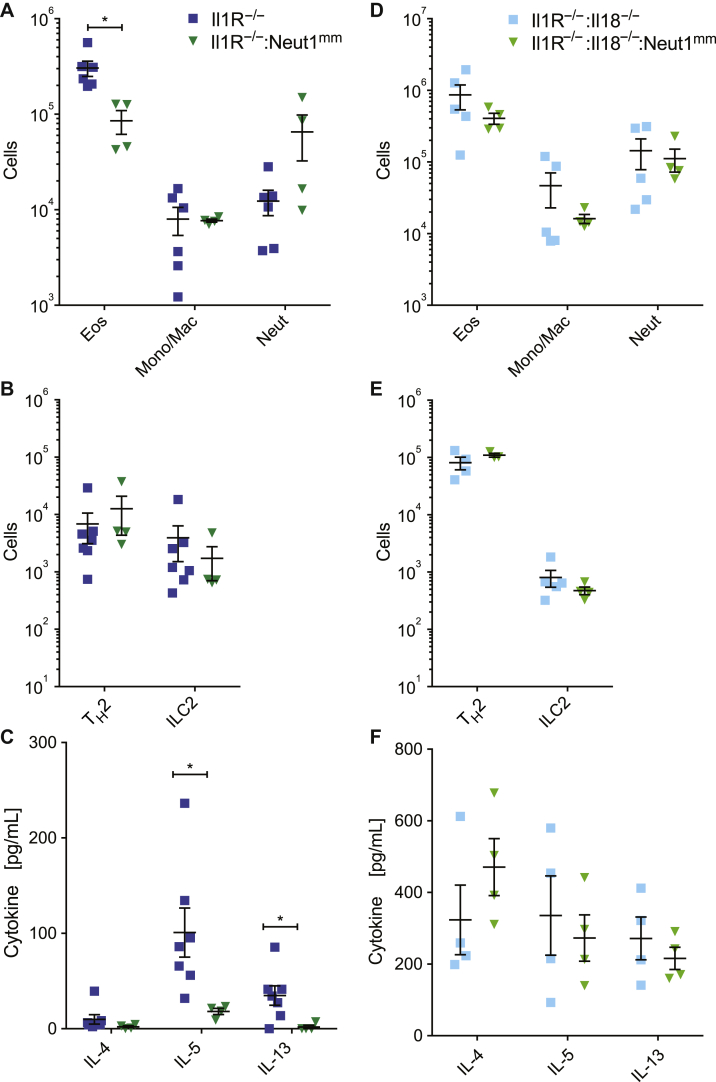
Protective effect of Nlrp1 in asthma model requires IL-18 signaling. Mice carrying an activating mutation of Nlrp1a (Q593P), referred to as *Neut1*^*m/m*^, are overtly healthy on the *Il1r*^*−/−*^ background, and studied compared with *Il1r*^*−/−*^ as control in the alum/OVA asthma model for (**A**) numbers of eosinophils (Eos), monocytes and macrophages (Mono/Mac), and neutrophils (Neut) in the lungs and (**B**) numbers of T_H_2 cells and ILC2s in the lungs. **C,** Cytokine levels measured in the BAL fluid. *Il1r*^*−/−*^*Il18*^*−/−*^*Neut1*^*m/m*^ mice were next investigated compared with *Il1r*^*−/−*^*/Il18*^*−/−*^ as control and studied in the alum/OVA asthma model for (**D**) numbers of eosinophils (Eos), monocytes and macrophages (Mono/Mac), and neutrophils (Neut) in the lungs and (**E**) numbers of T_H_2 cells and ILC2s in the lungs. **F,** Cytokine levels measured in the BAL fluid. *ILC2*, Type 2 innate lymphoid cell. Data are mean ± SEM. Fig 4, *A-C*, representative of 2 independent experiments. *P* values were calculated using unpaired *t* test between 2 groups. ∗*P* < .05.

The results obtained from *Nlrp1*^*−/−*^ mice suggest a protective effect of Nlrp1 activation in the asthma model. Therefore, we wanted to further investigate the role of the proinflammatory cytokines IL-1β and IL-18 in mediating protection during asthma, because both cytokines are released on Nlrp1 activation. To this end, we first looked at immune cell infiltration and cytokine levels in *Il1r*^*−/−*^ and *Il1r*^*−/−*^*/Neut1*^*m/m*^ mice during asthma. *Neut1*^*m/m*^ mice were described to have an Nlrp1a variant carrying an activating mutation (Q593P).^40^ Although *Neut1*^*m/m*^ mice have spontaneous lung inflammation, this is totally resolved in *Il1r*^*−/−*^*/Neut1*^*m/m*^ mice, which can be used in models of disease to establish effects that are independent of IL-1R. *Il1r*^*−/−*^*/Neut1*^*m/m*^ mice exhibited decreased eosinophilia in the lung during asthma (Fig 4, *A*), indicating a protective effect of NLRP1 activity even in the absence of IL-1R. This was further supported by a decrease in IL-5 and IL-13 levels in the BAL fluid of alum/OVA-treated *Il1r*^*−/−*^*/Neut1*^*m/m*^ mice compared with *Il1r*^*−/−*^ mice (Fig 4, *C*). Levels of monocytes/macrophages, neutrophils, T_H_2 cells, and ILC2s were comparable for both groups (Fig 4, *A* and *B*). IL-4 levels in the BAL fluid of both groups were also similar (Fig 4, *C*).

Because the protective effect of NLRP1 in asthma was independent of IL-1R signaling, we hypothesized that IL-18 plays a major role in mediating protection. We therefore investigated asthma pathology in *Il1r*^*−/−*^*Il18*^*−/−*^ and *Il1r*^*−/−*^*Il18*^*−/−*^*Neut1*^*m/*m^ mice. Again, we looked at immune cell infiltration into the lungs of these mice and at cytokine levels in the BAL fluid. Interestingly, there were no differences in eosinophilia or IL-5 and IL-13 cytokine levels observed for both groups (Fig 4, *D* and *F*). In addition, the levels of monocytes/macrophages, neutrophils, T_H_2 cells, and type 2 innate lymphoid cells were also comparable for both groups (Fig 4, *D* and *E*). These results indicate a role for IL-18 in mediating protection to asthma in an NLRP1-dependent manner.

## Discussion

The herein presented data are consistent with literature covering genetic associations of the inflammasome and IL-18 to asthma and further advances our understanding of the role of NLRP1 in this chronic disease.^10^, ^11^, ^12^^,^^22^^,^^51^^,^^52^ One limitation of our approach was to interrogate NLRP1 haplotypes that were previously established in vitiligo and other autoimmune disorders,^45^ so we may not have captured haplotype effects that were specific to asthma. In addition, a larger or more diverse asthmatic population than what we currently have available would be needed to replicate and validate these genetic associations. Our *in vivo* data establish that activation of Nlrp1a is protective in mouse models of this condition,^51^ via IL-18. Extrapolating from this, we provide evidence that the M1184V allele results in decreased inflammasome activation in the context of DPP9 inhibition. This would be consistent with the allele providing an increased disease risk, which we confirmed by genetic analysis of patients with asthma.

Our findings are novel because until now, studies looking at the role of M1184V did not activate the NLRP1 inflammasome with a specific stimulus. Using patient mutations that destabilize the N-terminus, or DPP9-inhibiting mutations/molecules, we are the first to observe that there is a dichotomous response, with the former resulting in increased inflammasome activation and the latter resulting in decreased inflammasome activation. Previously, it was documented that M1184V can increase autocatalytic cleavage of NLRP1,^21^ and that this could account for increased activation in the presence of N-terminal–destabilizing mutations. This is consistent with the observation that, where genetic information was available, all patients with dominantly inherited destabilizing N-terminal mutations have also possessed the M1184V allele, thus increasing NLRP1 activity.^24^ However, we now show that M1184V also increases binding to DPP9. Consequently, this would prevent its activation by DPP9 inhibition, which we also observed (Figs 1 and 2).

Dichotomous effects of M1184V on NLRP1 activation are also consistent with dichotomous effects in human diseases. Gathering all known disease associations for this allele, it is clear that a protective/causative role is not always consistent with inflammasome inhibition/activation, respectively (Table III).^53^, ^54^, ^55^, ^56^, ^57^, ^58^, ^59^, ^60^, ^61^, ^62^, ^63^, ^64^, ^65^, ^66^, ^67^, ^68^, ^69^, ^70^, ^71^, ^72^, ^73^, ^74^, ^75^, ^76^, ^77^, ^78^, ^79^, ^80^, ^81^, ^82^, ^83^, ^84^, ^85^, ^86^, ^87^, ^88^ Therefore, we suggest that in conditions such as asthma, where M1184V contributes to disease but NLRP1 has a protective role, the underlying mechanism of activation for NLRP1 resembles DPP9 inhibition. In contrast, for conditions in which M1184V and NLRP1 both contribute to disease, NLRP1 inflammasome activation would be mediated via destabilization of the N-terminus.

**Table III:** Diseases associated with the M1184V polymorphism

Disease	Odds ratio	*P* value	M1184V (rs11651270) contributes/protects to/from disease (PMID)	Inflammasome contributes/protects to/from disease (PMID)
Vitiligo and associated autoimmunity	1.6	NA	Contributes together with L155H/V1059M^45^ (23382179)	IL-1β contributes^53^^,^^54^ (28082234, 25221996)
Asthma	3.4	.013	Contributes^22^ (29154202)	IL-1β contributes^55^, ^56^, ^57^ (16210060, 23837489, 8527954) IL-18 protects^12^^,^^51^^,^^52^ (12006423, 10629451, 11972614)
Breast cancer	NA	.013	Potentially contributes^58^ (23107584)	IL-1β contributes^59^ (30545915) IL-18 protects^60^ (29725393)
HPV infection and associated cervical cancer	0.43	.003	Protects^61^ (26945813)	IL-1β contributes to cancer^62^ (19904560) IL-18 protects from infection^63^ (11470273)
Crohn disease	1.35	.02	Contributes to inflammatory phenotype^64^ (20403135)	IL-1β contributes^65^^,^^66^ (22891275, 7817982) IL-18 contributes^67^^,^^68^ (10352304, 10384110)
Chagas cardiomyopathy	NA	.036	Contributes^69^ (29438387)	IL-1β involvement unclear^70^ (30354432) IL-18 involvement unclear^71^ (25743241)
Type 1 diabetes	0.643	.002	Protects^72^ (31396539)	IL-1β unclear^73^^,^^74^ (23562090, 21518168) IL-18 contributes^75^^,^^76^ (25576800, 18359638)
Diabetic kidney disease	0.36	.01	Protects^77^ (29031829)	IL-1β contributes^78^^,^^79^ (27516236, 31191559) IL-18 contributes^80^, ^81^, ^82^ (12759891, 16306550, 17425653)
Malaria (*Plasmodium vivax*)	NA	NA	Potentially contributes^83^ (26946405)	IL-1β potentially contributes to severity^84^ (29602073) IL-18 reduces severity (with IL-12)^85^ (28615061)
Bacterial meningitis	2.32	.023	Potentially contributes^86^ (23053059)	IL-1β protects^87^ (12707352) IL-18 contributes to inflammation^88^ (12742650)

*NA*, Not applicable/available.

Previously, deletion of Nlrp1b was found to protect mice against inflammation in the lung due to inhalation of anthrax lethal toxin.^89^ This is consistent with the mechanism of that disease model being dependent on IL-1β and neutrophil influx. In contrast, our data to demonstrate that activation of Nlrp1 can prevent a model of asthma are consistent with results from a model of bleomycin-induced lung fibrosis.^90^ In that model, Talabostat (PT100) inhibition of DPP9 reduced collagen deposition and inflammation, which would agree with Nlrp1 activation providing a protective effect *in vivo*, presumably via IL-18.^91^ Given that insufficient NLRP1 exacerbated eosinophilia and IL-13 levels in the mouse model we studied, it is conceivable that blocking this axis with agents such as IL-4Rα may work more effectively in carriers of the NLRP1 M1184V variant. However, there are critical differences between NLRP1 in humans and mice, so the conclusions from our work need to be viewed in this context.

Collectively, this work defines the role of NLRP1 in asthma at a molecular level, and explains how the M1184V risk factor decreases activation in the context of DPP9 inhibition. This has implications for targeted therapies in asthma, and broader considerations for the NLRP1 inflammasome in other diseases associated with the M1184V allele.

**Key messages**

• NLRP1 SNP M1184V is associated with asthma.
• NLRP1 M1184V alters inflammasome formation depending on the method of activation.
• NLRP1 activation decreases severity of mouse asthma model.

## Figures and Tables

**Table E1:** GALA II Mexican American study participant demographics (discovery data set)

Characteristic	Asthma cases	Asthma controls	*P* value∗
N	610	295	†
Age (y), mean (SE)	12.6 (0.12)	15.0 (0.2)	2.20 × 10^−16^
Sex: female, %	42.60	59.30	3.45 × 10^−06^‡
Obese§ (N obese/N nonobese)	254/356	85/210	2.49 × 10^−04^‡

∗ Values presented were produced using the Wilcoxon rank-sum test unless otherwise indicated.

† Not determined.

‡ *P* value presented was generated using the χ^2^ test.

§ Obesity definition: Obese = BMI > 95th percentile; nonobese = BMI ≤ 95th percentile.

**Table E2:** GALA II Puerto Rican study participant demographics (replication data set)

Characteristic	Asthma cases	Asthma controls	*P* value∗
N	1078	340	†
Age (y), mean (SE)	12.5 (0.1)	12.9 (0.1)	.002
Sex: female, %	45.92	56.18	.0012‡
Obese§ (N obese/N nonobese)	320/758	78/262	.02‡

∗ Values presented were produced using the Wilcoxon rank-sum test unless otherwise indicated.

† Not determined.

‡ *P* value presented was generated using the χ^2^ test.

§ Obesity definition: Obese = BMI > 95th percentile; nonobese = BMI ≤ 95th percentile.

**Table E3:** SAGE African American study participant demographics (replication data set)

Characteristic	Asthma cases	Asthma controls	*P* value∗
N	877	379	†
Age (y), mean (SE)	14.1 (0.1)	16.2 (0.2)	2.20 × 10^−16^
Sex: female, %	48.35	57.52	.0035‡
Obese§ (N obese/N nonobese)	320/758	78/262	.0055‡

∗ Values presented were produced using the Wilcoxon rank-sum test unless otherwise indicated.

† Not determined.

‡ *P* value presented was generated using the χ^2^ test.

§ Obesity definition: Obese = BMI > 95th percentile; nonobese = BMI ≤ 95th percentile.

**Table E4:** Imputation quality control metrics

Variant ID	rs ID	Alleles	Imputation *R*^2^
R1366C	rs2137722	G/A	∗
†	rs6502867	T/C	∗
V1241L	rs11653832	C/G	∗
M1184T	rs146932154	A/G	0.98
M1184V	rs11651270	T/C	0.99
M1119V	rs35596958	T/C	∗
V1059M	rs2301582	C/T	∗
T995I	rs34733791	G/A	∗
T878M	rs11657747	C/A	0.99
T782S	rs52795654	G/C	0.99
T246S	rs11651595	G/C	0.99
L155H	rs12150220	A/T	0.99
†	rs2670660	A/G	0.99
†	rs8182352	C/T	0.99

∗ SNP was directly genotyped on the LAT1 genotyping array.

† No variant ID.

**Table E5:** SNP allele frequencies in Mexican Americans

Variant ID	rs ID	Effect allele	Alternate allele	Effect allele frequency		
				Asthma cases	Asthma controls	All participants
L155H	rs12150220	T	A	0.44	0.39	0.42
V1059M	rs2301582	T	C	0.42	0.36	0.40
M1184V	rs11651270	C	T	0.47	0.42	0.45

**Table E6:** SNP allele frequencies in Puerto Ricans

Variant ID	rs ID	Effect allele	Alternate allele	Effect allele frequency		
				Asthma cases	Asthma controls	All participants
L155H	rs12150220	T	A	0.29	0.30	0.30
V1059M	rs2301582	T	C	0.27	0.27	0.28
M1184V	rs11651270	C	T	0.42	0.40	0.42

**Table E7:** SNP allele frequencies in African Americans

Variant ID	rs ID	Effect allele	Alternate allele	Effect allele frequency		
				Asthma cases	Asthma controls	All participants
L155H	rs12150220	T	A	0.11	0.11	0.12
V1059M	rs2301582	T	C	0.14	0.15	0.14
M1184V	rs11651270	C	T	0.52	0.50	0.52

**Table E8:** NLRP1 haplotype definitions

Variant ID	rs ID	Alleles	NLRP1 haplotype definitions				
			1	1C	2A	2B	3
R1366C	rs2137722	G/A	**G**	G	G	G	A
∗	rs6502867	T/C	**T**	C	T	T	C
V1241L	rs11653832	C/G	**C**	C	C	C	G
M1184T	rs146932154	A/G	**A**	A	A	G	A
M1184V	rs11651270	T/C	**T**	T	C	C	C
M1119V	rs35596958	T/C	**T**	T	T	T	C
V1059M	rs2301582	C/T	**C**	C	T	T	C
T995I	rs34733791	G/A	**G**	G	G	G	A
T878M	rs11657747	C/A	**G**	G	G	G	A
T782S	rs52795654	G/C	**G**	G	G	G	C
T246S	rs11651595	G/C	**G**	G	G	G	C
L155H	rs12150220	A/T	**A**	A	T	T	T
∗	rs2670660	A/G	**A**	A	G	G	G
∗	rs8182352	C/T	**T**	T	C	C	C

Reference haplotype used for regression analyses is highlighted in boldface.

∗ No variant ID.

**Table E9:** NLRP1 haplotype frequencies by asthma status in Mexican Americans

Haplotype	Haplotype frequency		
	Asthma cases	Asthma controls	Overall
**Haplotype 1**	**0.31**	**0.38**	**0.33**
Haplotype 1C	0.17	0.15	0.17
Haplotype 2A	0.14	0.11	0.13
Haplotype 2B	0.25	0.22	0.24
Haplotype 3	0.01	0.02	0.01

Reference haplotype used for regression analyses is highlighted in boldface.

**Table E10:** NLRP1 haplotype frequencies by asthma status in Puerto Ricans

Haplotype	Haplotype frequency		
	Asthma cases	Asthma controls	Overall
**Haplotype 1**	**0.31**	**0.30**	**0.31**
Haplotype 1C	0.16	0.19	0.17
Haplotype 2A	0.17	0.17	0.17
Haplotype 2B	0.05	0.05	0.06
Haplotype 3	0.01	0.02	0.02

Reference haplotype used for regression analyses is highlighted in boldface.

**Table E11:** NLRP1 haplotype frequencies by asthma status in African Americans

Haplotype	Haplotype frequency		
	Asthma cases	Asthma controls	Overall
**Haplotype 1**	**0.35**	**0.35**	**0.35**
Haplotype 1C	0.04	0.04	0.04
Haplotype 2A	0.06	0.06	0.06
Haplotype 2B	0.007	0.008	0.008
Haplotype 3	0.02	0.02	0.02

Reference haplotype used for regression analyses is highlighted in boldface.

**Table E12:** NLRP1 haplotypes associated with asthma status in Puerto Rican children from the GALA II study

Haplotype	R1366C	rs6502867	V1241L	M1184T	M1184V	M1119V	V1059M	T995I	T878M	T782S	T246S	L155H	rs2670660	rs8182352	Odds ratio	95% CI	*P* value
Haplotype 1	G	T	C	A	T	T	C	G	G	G	G	A	A	T	Reference haplotype		
Haplotype 1C	G	C	C	A	T	T	C	G	G	G	G	A	A	T	0.84	0.64-1.10	.21
Haplotype 2A	G	T	C	A	C	T	T	G	G	G	G	T	G	C	0.96	0.74-1.25	.78
Haplotype 2B	G	T	C	G	C	T	T	G	G	G	G	T	G	C	0.91	0.60-1.36	.63
Haplotype 3	A	C	G	A	C	C	C	A	A	C	C	T	G	C	0.65	0.31-1.35	.24

**Table E13:** NLRP1 haplotypes associated with asthma status in African American children from the SAGE II study

Haplotype	R1366C	rs6502867	V1241L	M1184T	M1184V	M1119V	V1059M	T995I	T878M	T782S	T246S	L155H	rs2670660	rs8182352	Odds ratio	95% CI	*P* value
Haplotype 1	G	T	C	A	T	T	C	G	G	G	G	A	A	T	Reference haplotype		
Haplotype 1C	G	C	C	A	T	T	C	G	G	G	G	A	A	T	0.84	0.51-1.38	.49
Haplotype 2A	G	T	C	A	C	T	T	G	G	G	G	T	G	C	1.16	0.78-1.73	.45
Haplotype 2B	G	T	C	G	C	T	T	G	G	G	G	T	G	C	1.81	0.63-5.21	.27
Haplotype 3	A	C	G	A	C	C	C	A	A	C	C	T	G	C	0.83	0.42-1.65	.60

**Table E14:** NLRP1 variant association with asthma status in Puerto Rican children from the GALA II study

Variant	rs ID	Effect allele	Effect allele frequency	Odds ratio	95% CI	*P* value
L155H	rs12150220	T	0.30	0.94	0.78-1.14	.55
V1059M	rs2301582	T	0.28	1.00	0.83-1.21	.99
M1184V	rs11651270	C	0.42	1.04	0.88-1.24	.64

**Table E15:** NLRP1 variant association with asthma status in African American children from the SAGE II study

Variant	rs ID	Effect allele	Effect allele frequency	Odds ratio	95% CI	*P* value
L155H	rs12150220	T	0.12	1.05	0.78-1.40	.76
V1059M	rs2301582	T	0.14	0.99	0.77-1.28	.97
M1184V	rs11651270	C	0.52	1.08	0.91-1.29	.38
